# The effects of episodic context on memory integration

**DOI:** 10.1038/s41598-024-82004-7

**Published:** 2024-12-04

**Authors:** Zhenghao Liu, Mikael Johansson, Roger Johansson, Inês Bramão

**Affiliations:** Department of Psychology, Lund University, BOX 213, 221 00 Lund, Sweden

**Keywords:** Episodic memory, Memory integration, Associative inference, Episodic context, Flexible retrieval, Integrative encoding, Psychology, Human behaviour

## Abstract

Information encountered in different events, such as people and objects, can be interlinked in memory. Such memory integration supports novel inferences about the world. This study investigates the role of episodic context on memory integration in two experiments using an associative inference task. Participants encoded events with overlapping (AB and BC) and non-overlapping associations (XY) presented in the same or different episodic contexts. Inference performance across events (AC) was tested in the absence (Experiment 1) and in the presence (Experiment 2) of the encoding context. Our data show that inferences across events encoded in the same context were more accurate, faster, and made with greater confidence compared to those encoded in different contexts. However, this effect was observed only when the context was presented during testing, suggesting that context enhances associative inferences by facilitating retrieval of events associated with that context. These findings demonstrate that revisiting the encoding context promotes memory integration by providing privileged access to contextually associated memory traces and facilitating their flexible recombination to form novel inferences.

**Supplementary Information:**

The online version contains supplementary material available at 10.1038/s41598-024-82004-7.

## Introduction

Episodic memory enables mental time travel into our personal past and future^1^. Memories can extend beyond direct experience by linking overlapping information encountered at different times and places^2,3^. For example, encountering your colleague and her daughter in the park might remind you of seeing the same child playing with a man in the park the previous week. By integrating the memories of these two events, you may infer that your colleague and the man are somehow related. Such inferences are thought to rely on memory integration processes and are crucial to support novel decisions and extend knowledge to entirely new situations^4,5^. This study investigates whether the episodic context influences the integration of memories across events and examines how different underlying memory integration mechanisms are affected.

Memory integration is usually investigated with the associative inference paradigm^6–8^. Participants are presented with pairs of associates composed of two elements (AB) followed by another set of associates containing an overlapping element (BC). In the subsequent memory test, participants are asked to infer the AC relationship via the overlapping element B. Successful performance on the final AC inference can be explained by at least two complementary accounts. The integrative encoding account posits that the related AB associate is retrieved during BC encoding, which leads to the formation of an integrated ABC memory representation used to later infer the AC relationship^9^. The flexible retrieval account^10^ proposes that the AB and the BC associates are encoded and stored separately and that the AC link is created by flexibly recombining these two distinct memory representations at the time of the test^10,11^. Although the two accounts suggest that links between events are established at different time points, both emphasize that inferences result from the interaction between past and new events^7,12–14^.

Returning to an encoding context can serve as a powerful retrieval cue^15,16^. The seminal study by Godden and Baddeley^15^ first demonstrated that episodic remembering improves when the encoding context is revisited during retrieval. This context reinstatement benefit has been supported by a large body of memory research^16^ highlighting the context dependency of episodic remembering^17,18^. Context dependency is commonly explained by the encoding-specificity principle, which suggests that greater overlap between encoding and retrieval contexts enhances episodic remembering^19,20^. Consequently, we predicted that revisiting a familiar context could promote the retrieval of previous events, facilitating the interaction between new and old events and supporting associative inference processes.

However, this prediction conflicts with recent theories stating that context similarity across related events can create memory interference^21^. Accordingly, context similarity may impair encoding and retrieval when irrelevant memory traces are reactivated with relevant memories, thereby creating interference^22,23^. This notion is further supported by the well-stablished fan effect^24,25^, which demonstrates that memory interference increases as the number of associates, or ‘fan’, linked to a cue grows. The more associations connected to a single context, the greater the competition between memory traces, making it more difficult to accurately retrieve specific memories^24^. Thus, competition between events associated with the same episodic context may complicate the formation of associative inferences by enhancing interference during encoding and retrieval of events with overlapping content.

Cox et al.^26^ examined how episodic context modulates the interactions between memory interference and memory inference and provided evidence supporting the idea that the episodic context promotes the integrative encoding processes involved in making inferences. They found that when events are encoded in the same context, the potential competition brought by the same context is resolved by creating an integrated memory representation that incorporates elements from past and new events. This integrated memory representation reduces interference and facilitates later inferences across events occurring in the same context. Neuroimaging studies have provided further support for this idea. For example, in a recognition memory task, Libby and colleagues^27^ showed that brain patterns differentiate between events sharing either elements or context information but integrate across events that share the same elements and context.

Despite these insights, previous studies have typically manipulated context in an event-specific fashion with one unique context per encoding event. Importantly, however, current neurobiological models of memory posit that episodic memories result from binding discrete events to a slowly drifting context^28–31^. Thus, when investigating the role of context in episodic memory processes, it is essential to use experimental paradigms where the context manipulation is incidental to the task and more stable over time^16,32,33^. The present study investigates the role of episodic context in memory integration using a context manipulation that adheres to these principles.

**Fig. 1:**
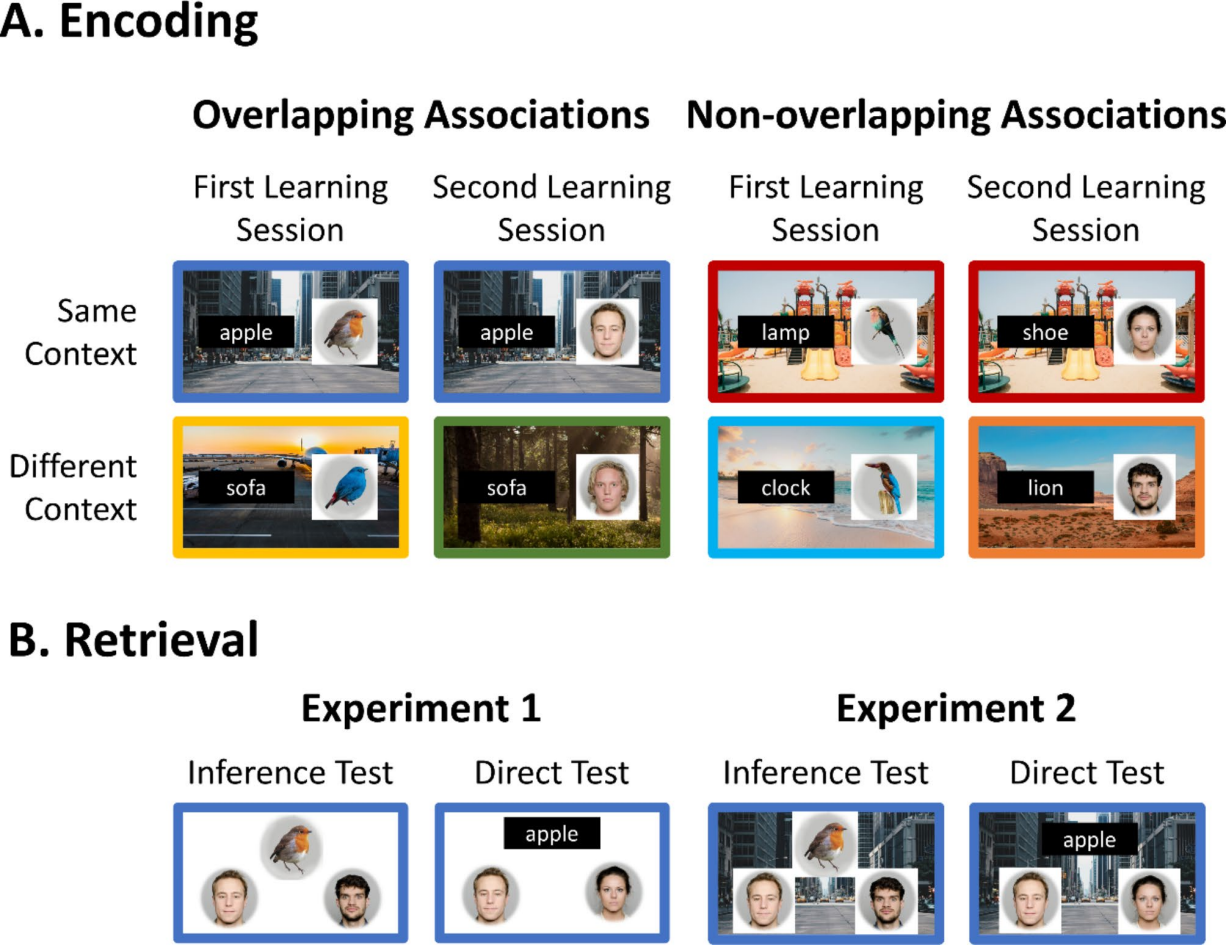
Experimental design. (**A**) Encoding phase. Participants encoded events with overlapping (i.e., ABs and BCs) and non-overlapping (i.e., XYs) associations across the same and different background contexts. To make context stable over time, the background context was shared across multiple events within the same experimental condition. (**B**) Retrieval phase. Experiment 1 and 2 tested, respectively, AC inference performance in the absence and in the presence of the encoding context. In Experiment 2, when the events were encoded in different contexts, the AC inference was tested in the presence of the context associated with the cue, that is the element appearing on top of the screen. For the inference test, the cue is a picture and for the direct association test, the cue is a word. After the AC inference test, performance for the direct events (i.e., ABs, BCs and XYs) was also tested. The faces were selected from the Oslo Face Database^34^ The individuals displayed have provided permission for publication of their image. The birds and the contexts are similar to the ones used in the original experiments; however, for illustrative purposes we used license-free pictures from the unsplash data base (https://unsplash.com/license).

Memory integration was measured with an adapted associative inference paradigm^6–8^. Participants encoded events comprising two elements, a word and a picture, superimposed on a background photo, serving as the episodic context. We included events with overlapping elements, a shared word (i.e., AB and BC), and events without overlapping elements (i.e., XY). We contrasted inference performance for events with overlapping elements (i.e., AB and BC) encoded in the same versus different episodic contexts. Additionally, we measured inference performance for events with non-overlapping elements (i.e., XY). Inference performance for these events can only be driven by the same episodic context, as they do not have overlapping elements (see Fig. 1A).

To create a stable context experience, several paired associates were presented in the same context consecutively^33^. Critically, this paradigm adheres to the principles of context established in the current neurobiological models of memory^28–31^ and mimics everyday experiences where multiple events occur in the same context. This enables us to disentangle the specific roles of episodic context and other event elements, such as the overlapping B element.

In Experiment 1, the associative inference test was conducted without the encoding context, whereas in Experiment 2, the encoding context was present at test (see Fig. 1B). Context may boost inference performance by enhancing the integrative encoding processes involved in inference-making. That is, revisiting the same context during BC learning can promote the reactivation of previous overlapping AB association encoded in that context. This reactivation may boost the creation of an integrated ABC memory trace, which is then retrieved during the AC inference test phase, resulting in more accurate and faster associative inferences for events occurring in the same context. Additionally, context may promote associative inference by facilitating the flexible retrieval processes supporting inferences at test. The presence of context during the test phase may promote retrieval of the events encoded in that context, enabling their recombination to infer indirect associations.

By contrasting the potential benefits of context across Experiments 1 and 2, we aim to elucidate the mnemonic processes involved in memory integration that are potentially facilitated by the episodic context. If a shared context promotes integrative encoding, we expect to observe a shared context benefit in associative inference performance in both experiments. If context also promotes the flexible recombination of the events at retrieval, the shared context benefit should be higher in Experiment 2 compared with Experiment 1. Alternatively, if context only promotes associative inference by facilitating the flexible recombination at retrieval, a shared context benefit is expected in Experiment 2 but not in Experiment 1.

## Experiment 1

Experiment 1 investigated if associative inferences across events encoded in the same context are easier to make compared with inferences across events encoded in different contexts. The encoding context was not presented at retrieval.

### Method

#### Participants

To comply with the sample sizes of the previous literature^26^, we aimed to recruit at least 50 participants. Since no prior power analysis was conducted, we performed a sensitivity power analysis aiming to find the smallest effect size that could be detected with our design (see Data Analysis). Data were collected using an online platform (https://www.prolific.com). Because of the uncertainties of online data collection, we recruited 65 participants. Participants provided informed consent and were monetarily compensated, £7.5 per hour, according to the Prolific recommendations. The data collection was anonymous and did not involve any potentially identifying demographic information. The data collection was conducted in accordance with the Swedish Act concerning the Ethical Review of Research involving Humans (2003:460) and the Code of Ethics of the World Medical Association (Declaration of Helsinki). As established by Swedish authorities and specified in the Swedish Act concerning the Ethical Review of Research involving Humans (2003:460), the present study does not require specific ethical review by the Swedish Ethical Review Authority due to the following reasons: (1) it does not deal with sensitive personal data, (2) it does not use methods that involve a physical intervention, (3) it does not use methods that pose a risk of mental or physical harm, (4) it does not study biological material taken from a living or dead human that can be traced back to that person. Additionally, the Ethics Committee at the Department of Psychology, Lund University, has corroborated that the present research protocol follows the research ethics guidelines established by Swedish authorities.

Assuming that the context manipulation is ineffective for those participants who perform either very poorly (below chance level) or exceptionally well (ceiling effect) on the AC inference task, we excluded six participants due to low accuracy in the AC memory inference test (accuracy lower than 40%, which corresponds to performance significantly lower than the 50% chance level) and another three due to ceiling performance (above 90%). As such, the final sample comprised data from 56 participants (29 female, 27 males, *M*_*age*_ ± *SD* = 25.39 ± 4.97).

#### Material

The ABC overlapping associates consisted of 24 triplets formed by a word (B), a picture of a face and a picture of a bird (A and C). For half of the associates, the A was a face, the C was a bird, and the other half was the reverse. The XY non-overlapping associates comprised 72 words paired with a picture of a face or a bird. The words consisted of 96 common nouns. The faces consisted of 60 pictures of female faces selected from the Oslo Face Database^34^ and the birds were 60 pictures previously used in a similar study^35^. The pictures of faces and birds were resized to 500 × 526 pxl. The paired associates were displayed superimposed on contextual background pictures.

The contextual background pictures were 48 photos, including indoor and outdoor environments^22^, resized to 1024 × 576 pxl, but magnified to fill the whole screen when presented.

The words were divided into eight equivalent lists, matched in frequency and size. Four lists were randomly assigned to the three main experimental conditions: (a) events with overlapping associations encoded in the same context, (b) events with overlapping associations encoded in different contexts, and (c) two lists for the events with non-overlapping associations encoded in the same context. The remaining four lists were distributed across the events with non-overlapping associations encoded in different contexts. Similarly, the contextual background photos were randomly divided into eight lists and assigned to each experimental condition. To ensure that differences between conditions are not due to differences in the material, the assignment of the lists was counterbalanced across experimental conditions and participants.

#### Procedure

Participants signed up on Prolific and were then redirected to Pavlovia (https://pavlovia.org/) where the experiment was presented. The experiment comprised six blocks. The complete procedure took about an hour and participants were encouraged to take a break between each block. Each block comprised an encoding phase and a test phase, separated by a distractor task. In the encoding phase of each block, participants were presented with four AB and six XY pairs, followed by the presentation of four BC and another six XY pairs. The presentation of the ABs and BCs was intermingled with the presentation of the XYs. Two BC pairs were presented in the same context as the previous AB pairs. The other two were presented in a new context. Two of XY pairs were presented in the same context as two of the previous XYs. The remaining XY pairs were presented in two new contexts. Including more XYs than ABs and BCs permitted us to use XYs, presented in different contexts, as distractors for the memory tests. Our manipulation of context followed the principles established in current neurobiological models of memory^28–31,33^; that is, each event occurred superimposed on a background context that was more stable over time. Two paired associates of the same condition were presented consecutively in the same background contextual photo.

Participants were informed they would encounter several different word-picture associates in different contexts. Their task was to memorize all associations and simultaneously establish indirect links between faces and birds through the overlapping word and/or context. The presentation order of all conditions within a block was counterbalanced across blocks and participants.

In each block, there were a total of 20 learning trials (four ABs and six XYs in the first learning session, and 4 BC and six XYs in the second learning session). Each learning trial started with the presentation of a fixation cross for 1s, followed by the context for 1.5s. After this, the paired associates were displayed on top of the context and remained on the screen for 6s. The test phase was preceded by a 1-minute distraction task, requiring participants to consecutively subtract 7 from a random 3-digit number. During the test phase, participants were asked to select the picture that was directly or indirectly linked to a cue. First, all six indirect associations were tested (four ACs and two XYs). The cue—a picture of a face or a bird—was displayed on top of a black screen for 1s. The target and distractor were then displayed and remained on the screen for 10s. The target was the corresponding bird or face indirectly linked with the cue through the overlapping word and/or the same encoding context. The distractors were selected from the non-overlapping associations (XY) presented in different contexts and were from the same learning phase (the first vs. the second learning session) and category (face vs. bird) as the targets. Participants pressed either the left or the right arrow to indicate their response. The positions of the target and distractor were counterbalanced across conditions. After each inference test, participants rated their confidence using the number keys, where 1 = guessing; 2 = maybe and 3 = sure.

Following all the AC inference tests, the 20 direct associations were examined. The procedure was identical to the inference test, with the only difference being that the cues were the B/X words. For half of the overlapping associations, the AB pairs were tested first, followed by the BC pairs. For the other half, the order was reversed. The test of the XY pairs was intermingled with the test of the AB and BC pairs.

#### Data analysis

Data were analyzed in R (4.1.2). Differences in memory retrieval across conditions were tested using linear mixed models. The packages of *lme4* (1.1–34) and *emmeans* (1.8.7) were used to fit the models, perform post-hoc tests, and get estimated marginal means for each condition. The first level of each model was the trial level, which was clustered in the second level, i.e., the participant level. To rule out potential confounds of random effect^36^ and simultaneously prevent model overfitting^37^, the fitting of each model started with participants as the only random intercept. Other factors were thereafter added as random slopes in a step-by-step fashion. If an added random slope improved the model fitting significantly (i.e., *p* < .05 in chi-square test for model fitting comparison, decreased the Akaike Information Criterion and Bayesian information criterion), the random slope was kept. In Supplementary Note 1, it is possible to consult the final equations for all models, showing which random factors were included for each analysis. For each model, the homogeneity of variance of the residuals was assessed by using Levene’s test. If the test indicated heteroskedasticity, we re-ran the model with a restricted variance structure^38^. Significant interactions were followed up with Tukey-corrected post-hoc tests. Effect sizes are reported together with the statistics. In the mixed models we report the $\:{\eta\:}_{p}^{2}$ and for the post hoc *t-*tests we report the unstandardized difference *D*.

Memory retrieval was examined using accuracy, response time, and confidence in correct responses. Response times were calculated from the onset of the test probe until the participants’ response. Because response times did not follow a normal distribution, they were logarithmic transformed.

We examined the effects of context on the associative inference test. In our experiment, associative inference could be made through the overlapping B element and/or through the same context. Thus, associative inference performance was contrasted across three different conditions: ABC encoded in the same context vs. ABC encoded in different contexts vs. XY encoded in the same context. A sensitivity power analysis was performed using the package of *mixedpower* (0.1.0). Due to the complexity of the covariance structure of the linear mixed models, the power analysis was performed via data simulation^39^. A simulated dataset was generated based on a designated effect size and the design matrix of the present experiment, i.e., number of trials for each condition nested in the total number of participants. A series of effect sizes was tested, with each effect size undergoing 500 iterations of simulation to estimate its statistical power. Starting with an initial effect size, the value was iteratively adjusted based on the estimated power. If the resulting power was below 80%, the effect size was incrementally increased and tested again; if the power exceeded 80%, the effect size was reduced. This iterative procedure continued until an effect size was identified that achieved a statistical power of exactly 80%, accurate to two decimal places, with alpha set to 5%. This simulation-based sensitivity analysis showed that the smallest effect size that we could capture in the present experiment for the inference performance analysis was $\:{\eta\:}_{p}^{2}$ = 4.06e-3. As a reference, the effect size observed in Cox et al.’s study^26^ was $\:{\eta\:}_{p}^{2}$ = 0.38 (converted from *z* = 5.307, with 48 participants).

Next, we explored the effects of context on the retrieval of the direct associations, considering the factors Context (Same vs. Different) and Association Type (ABC vs. XY). Same Context refers to paired associates (ABC and XY) that have been encoded in a shared context across the first and the second learning sessions. On the other hand, Different Context refers to paired associates (ABC and XY) that have been encoded in different contexts across the first and the second learning sessions. The simulation-based sensitivity power analysis revealed an 80% power of detecting fixed effect of $\:{\eta\:}_{p}^{2}$ = 6.65e-4, using an alpha level of 5%.

To provide evidence for non-significant findings critical to our interpretations, we used a Bayesian approach. Unlike frequentist statistics, which can only result in not rejecting the null hypothesis, Bayesian analysis allows for measuring the strength of evidence supporting it. The package of *BayesFactor* (0.9.2+) was used for this aim. The differences were compared using a t-test with a Cauchy distribution prior (gamma = 0.707). Bayesian Factors, in favor of null hypothesis and against alternative hypothesis, i.e., BF_01_, larger than 3 were considered evidence for the null hypothesis. On the other hand, a BF_01_ between 0.3 and 3 indicates that no conclusions can be drawn^40^. The Bayesian Factors are reported together with the 95% credible interval of the posterior, using the MCMC method^41^.

## Results

### Associative inference

**Fig. 2:**
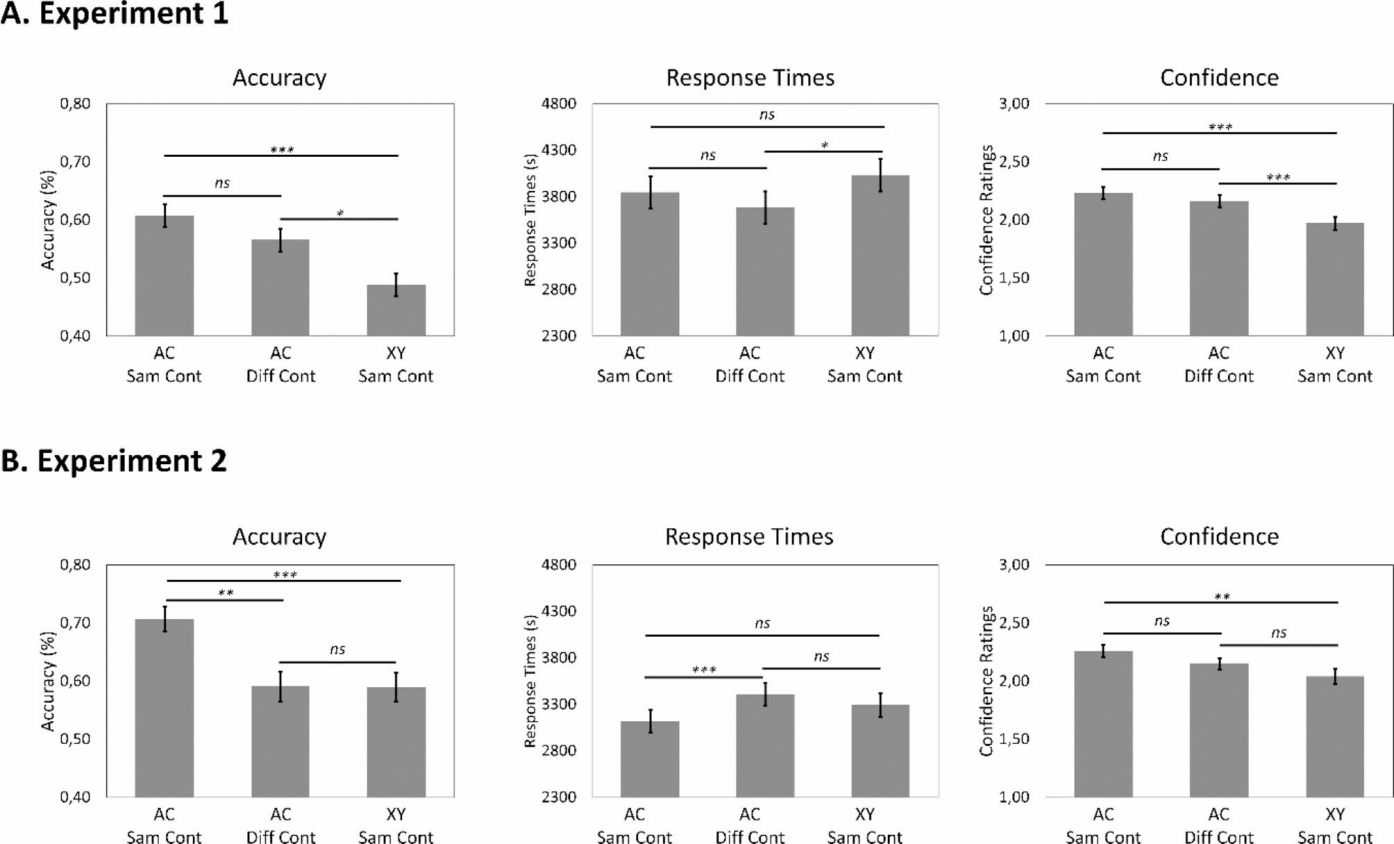
Associative inference performance in terms of accuracy, response times, and confidence ratings for Experiment 1 (**A**) and Experiment 2 (**B**). The error bars represent the standard errors of estimated marginal means. Statistical comparisons of interests are marked (*** for *p* ≤ .001; ** for *p* ≤ .01; * for *p* ≤ .05 and *ns* for *p* > .05).

Associative inference performance was compared across the three experimental conditions: events with overlapping ABC associations encoded in the same context vs. events with overlapping ABC associations encoded in different contexts vs. events with non-overlapping XY associations encoded in the same context. The data showed significant effects of condition across all memory measures (accuracy: *F*(2,1960) = 10.088, *p* < .001, $\:{\eta\:}_{p}^{2}$ = 0.01; response times: *F*(2,1063) = 4.144, *p* = .016, $\:{\eta\:}_{p}^{2}$ = 0.01; confidence: *F*(2,1066) = 14.449, *p* < .001, $\:{\eta\:}_{p}^{2}$ = 0.03; see Fig. 2). Post-hoc tests showed that participants were more accurate, faster, and confident at making associative inferences for events with overlapping ABC associations compared with events with non-overlapping XY associations (all *p*s < 0.012). Crucially, however, there were no differences in the inference performance for events with overlapping ABC associations encoded in the same and in different contexts (accuracy: *t*(1962) = 1.548, *p* = .269, *D* = 0.042; response times: *t*(1065) = 1.642, *p* = .228, *D* = 0.032; confidence: *t*(1068) = 1.416, *p* = .333, *D* = 0.067; see Fig. 2), providing no evidence that the same encoding context promotes associative inference performance. To provide evidence for these null results, we employed a Bayesian approach. Critically, the Bayesian analysis provided evidence for comparable associative inference performance for events with overlapping elements encoded in the same and in different contexts (accuracy: *BF*_*01*_ = 4.808, 95% CI of posterior = [-0.006. 0.096]; response times: *BF*_*01*_ = 14.286, 95% CI of posterior = [-0.029. 0.049]; confidence: *BF*_*01*_ = 4.902, 95% CI of posterior = [-0.032. 0.160]; see Fig. 2).

Moreover, a one-sample *t*-test showed that inference performance for events with non-overlapping XY associations encoded across the same context was not different from the 50% chance level (*t*(357) = -0.603, *p* = .547, *D* = -0.012), suggesting that context alone does not promote associative inferences. The Bayesian approach provided supportive evidence for this null result (*BF*_*01*_ = 19.231, 95% CI of posterior = [-0.049. 0.028]).

### Direct associations

**Fig. 3:**
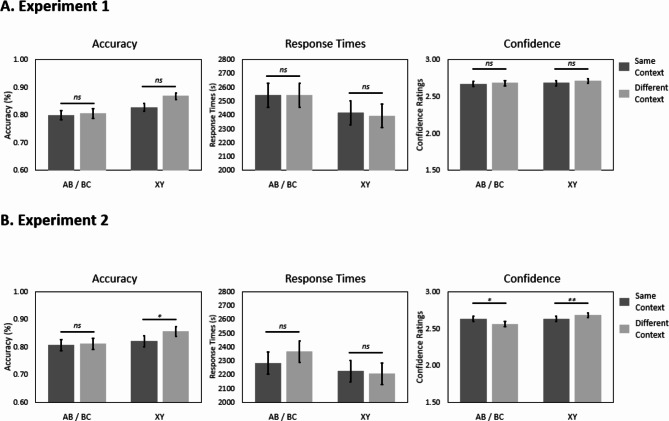
Performance for the direct associations in terms of accuracy, response times, and confidence for overlapping (AB and BC) and non-overlapping (XY) associations encoded in the same and in different contexts for Experiment 1 (**A**) and Experiment 2 (**B**). Error bars indicate the standard errors of estimated marginal means. Statistical comparisons of interests are marked (*** for *p* ≤ .001; ** for *p* ≤ .01; * for *p* ≤ .05 and *ns* for *p* > .05).

Next, we investigated the effects of context on the memory performance for the direct associations. We observed that non-overlapping XY associations were retrieved more accurately and faster compared with overlapping AB/BC associations (accuracy: *F*(1,67) = 15.550, *p* < .001, $\:{\eta\:}_{p}^{2}$ = 0.19; response times: *F*(1,5545) = 18.375, *p* < .001, $\:{\eta\:}_{p}^{2}$ = 3.30e-3; confidence: *F*(1,5545) = 2.631, *p* = .105, $\:{\eta\:}_{p}^{2}$ = 4.74e-4; see Fig. 3). Additionally, a significant effect of context was observed (accuracy: *F*(1,57) = 4.245, *p* = .044, $\:{\eta\:}_{p}^{2}$ = 0.07; response times: *F*(1,5545) = 0.348, *p* = .555, $\:{\eta\:}_{p}^{2}$ = 6.28e-5; and confidence: *F*(1,5545) = 1.537, *p* = .215, $\:{\eta\:}_{p}^{2}$ = 2.77e-4; see Fig. 3), showing that performance was lower for associations presented in the same context (i.e., shared with a different pair of associates) across the two learning phases, compared with associations encoded in a different context (i.e., not shared with a different pair of associates) across the two learning phases. No significant interactions between association type and context were observed (accuracy: *F*(1,64) = 2.310, *p* = .133, $\:{\eta\:}_{p}^{2}$ = 0.03; response times: *F*(1,5545) = 0.114, *p* = .736, $\:{\eta\:}_{p}^{2}$ = 2.05e-5; confidence: *F*(1,5545) = 0.429, *p* = .513, $\:{\eta\:}_{p}^{2}$ = 7.74e-5).

### Summary

Experiment 1 tested the prediction that associative inferences across events are facilitated when events are encoded in the same context. We reasoned that if a familiar context facilitates access to previously encoded memory traces^15,16^, encoding novel information within this familiar context would enhance the likelihood of past events interacting with new information. This would consequently lead to the formation of an integrated memory representation that could be later utilized at test to make the AC inference. However, our data did not support this prediction. Inference performance was comparable for events that were encoded in the same and in different contexts. Moreover, inference performance for events with non-overlapping elements encoded in the same context did not significantly differ from chance, suggesting that simply encoding in the same context is not sufficient to promote associative inference. Critically, the Bayesian approach provided evidence suggesting that the episodic context does not promote associative inference by boosting integrative encoding processes.

Furthermore, we also observed that memory retrieval for the direct associations was poorer for overlapping compared with non-overlapping associations and for associations encoded in the same compared with different contexts. These results suggest that similarities across events during encoding, driven by either content or context, are likely associated with memory interference brought by the overlap between the different memory traces^22,23,42^.

## Experiment 2

Experiment 1 showed no evidence that the encoding context promotes associative inferences, at least not via boosting the formation of an integrated memory trace during encoding. Experiment 2 investigated if the context promotes associative inferences by boosting the flexible retrieval processes involved in making inferences. Context may promote associative inferences by providing privileged access to the events associated with a given context at the time of testing. To investigate this prediction, the encoding context was presented at the time of testing.

### Methods

#### Participants

Data for Experiment 2 were collected in the same way as in Experiment 1. Again, we aimed for at least 50 participants with usable data. No prior power analysis was conducted; however, we performed a sensitivity power analysis aiming to find the smallest effect size that could be detected with our design (see Data Analysis). Due to uncertainties with online data collection, we recruited data from 61 participants. Participants gave their informed consent and were compensated monetarily, £7.5 per hour, according to the Prolific recommendations, for their participation. The data collection was anonymous so no potentially identifying demographic information was included. This experiment followed the same ethical considerations as Experiment 1.

The data of five participants were excluded due to low average memory performance on the AC inference task (accuracy lower than 40%, which corresponds to performance significantly lower than the 50% chance level) and the data of two participants were excluded due to ceiling performance (associative inference task accuracy higher than 90%). The final sample comprised 54 participants (28 female and 26 male, *M*_*age*_ ± *SD* = 25.11 ± 4.93).

#### Material and procedure

This experiment used the same stimuli material and procedure as in Experiment 1. The only difference is that the encoding context was presented at test, together with the cue. In the different context condition the AC inference was tested in the presence of the context where the cue (A or C) was encoded.

### Data analysis

The data was analyzed in the same way as for Experiment 1. The random slopes included in the final models are provided in Supplementary Note 1. Simulation-based sensitivity power analyses^39^ were also performed for the present experiment with the package of *mixedpower* (0.1.0), considering an alpha level of 5% and power of at least 80%. The analysis showed that the smallest effect sizes we could detect were $\:{\eta\:}_{p}^{2}$ = 4.57e-3 for the associative inference analysis and $\:{\eta\:}_{p}^{2}$ = 8.43e-4 for the direct associations analysis.

## Results

### Associative inference

Associative inference performance was contrasted across three conditions: events with overlapping ABC associations encoded in the same context vs. different contexts vs. events with non-overlapping XY associations encoded in the same context. We observed significant effects in all dependent measures (accuracy: *F*(2,92) = 13.150, *p* < .001, $\:{\eta\:}_{p}^{2}$ = 0.22; response times: *F*(2,1173) = 6.907, *p* = .001, $\:{\eta\:}_{p}^{2}$ = 0.01 and confidence: *F*(2,54) = 8.193, *p* < .001, $\:{\eta\:}_{p}^{2}$ = 0.23; see Fig. 2). Critically, participants were better and faster at making associative inferences when the overlapping ABC events were encoded in the same context compared with different contexts (accuracy: *t*(54) = 3.955, *p* = .001, *D* = 0.116; response time: *t*(1175) = 3.643, *p* = .001, *D* = 0.049). The same tendency was also found for the confidence analysis (*t*(51) = 2.219, *p* = .077, *D* = 0.114).

The accuracy for associative inferences based on events with non-overlapping XY associations, thus solely based on their shared context, was significantly above chance level (*t*(55) = 3.645, *p* = .001, *D* = 0.090). Inference performance in this condition was comparable with inference performance for events with overlapping ABC associations encoded in different contexts (accuracy: *t*(55) = -0.038, *p* = .999, *D* = 0.002; response times: *t*(1178) = -1.300, *p* = .3956, *D* = -0.018; confidence: *t*(54) = -1.376, *p* = .3607, *D* = -0.101), but lower when compared with inference performance for events with overlapping ABC associations encoded in the same context (accuracy: *t*(54) = 3.692, *p* = .002, *D* = 0.117; confidence: *t*(54) = 3.583, *p* = .002, *D* = 0.215). The same tendency was also observed for response times (*t*(1175) = -2.269, *p* = .061, *D* = -0.030).

### Direct associations

Next, we investigated the context effects on memory performance for the direct associations. We observed significant effects of association type (accuracy: *F*(1,6426) = 9.934, *p* = .002, $\:{\eta\:}_{p}^{2}$ = 1.54e-3; response times: *F*(1,5324) = 14.486, *p* < .001, $\:{\eta\:}_{p}^{2}$ = 2.71e-3; confidence: *F*(1,6077) = 19.253, *p* < .001, $\:{\eta\:}_{p}^{2}$ = 3.16e-3), revealing that participants were more accurate, faster and confident at retrieving non-overlapping XY associations compared with overlapping AB/BC associations. See Fig. 3. The effect of context was significant in the accuracy analysis (accuracy: *F*(1,6426) = 5.342, *p* = .021, $\:{\eta\:}_{p}^{2}$ = 8.31e-4; response times: *F*(1,5324) = 2.245, *p* = .134, $\:{\eta\:}_{p}^{2}$ = 4.22e-4; and confidence: *F*(1, 6078) = 0.010, *p* = .923, $\:{\eta\:}_{p}^{2}$ = 1.56e-6), and showed that associations encoded in the same context (i.e., shared with a different pair of associates) across the two learning sessions were more difficult to retrieve than associations encoded in different contexts (i.e., not shared with a different pair of associates).

Finally, we found a significant interaction between the two factors in the confidence model (accuracy: *F*(1,6426) = 2.418, *p* = .120, $\:{\eta\:}_{p}^{2}$ = 3.76e-4; response times: *F*(1,5324) = 2.922, *p* = .087, $\:{\eta\:}_{p}^{2}$ = 5.48e-4; and confidence: *F*(1,6077) = 13.893, *p* < .001, $\:{\eta\:}_{p}^{2}$ = 2.28e-3). The post-hoc tests revealed that overlapping ABC pairs were more confidently retrieved when encoded in the same context across the two learning sessions compared with a different context (*t*(6081) = 2.516, *p* = .012, *D* = 0.056). However, non-overlapping XY pairs encoded in the same context across the two learning sessions were retrieved less confidently compared with those encoded in different contexts (*t*(6080) = -2.790, *p* = .005, *D* = -0.053).

### Summary

Experiment 2 tested if episodic context promotes associative inferences by boosting the flexible retrieval processes involved in creating inferences across events. Our data clearly showed that associative inference is benefited for events encoded in the same context when context is present at test. Moreover, presenting the encoding context at test enabled inferences for events with non-overlapping XY associations solely driven by the shared context. In sum, presenting the encoding context at retrieval promotes associative inference performance by facilitating access to the memory traces there encoded, which facilitates the linking between the memories.

Additionally, in alignment with what was found in Experiment 1, retrieval performance for the direct overlapping associations was lower compared with non-overlapping associations. This corroborates previous research^22,23^ and shows that when memory traces overlap, memory suffers from interference brought by the memory traces similarity.

## Experiment 1 vs. experiment 2

Finally, we quantify the benefit brought by presenting the encoding context at the time of test by directly contrasting memory performance for Experiments 1 and 2. First, inference performance was contrasted across Experiments using linear mixed models with the between-subject factor Experiment (Experiment 1 vs. Experiment 2) and the within-subject factor Experimental Condition (ABC encoded in the same context vs. ABC encoded in the different context vs. XY encoded in the same context). Next, we contrasted performance for the direct associations, also with linear mixed models with Experiment (Experiment 1 vs. Experiment 2) as a between-subject factor and the within-subject factors Context (Same vs. Different) and Association Type (Overlapping AB/BC vs. Non-overlapping XY). We used the same simulation-based power analysis to investigate the smallest effect size that we could detect in this analysis. Considering an alpha level of 5% and a power above 80%, the smallest effect size that we could detect was $\:{\eta\:}_{p}^{2}$ = 2.12e-3 for the associative inference analysis and $\:{\eta\:}_{p}^{2}$ = 1.46e-3 for the direct associations analysis.

### Associative inference

This analysis contrasted inference performance for Experiments 1 and 2 across the three experimental conditions: events with overlapping ABC associations encoded in the same context vs. different contexts vs. events with non-overlapping XY associations encoded in same context. We found significant main effects of condition in all dependent measures (accuracy: *F*(2,178) = 18.824, *p* < .001, $\:{\eta\:}_{p}^{2}$ = 0.17; response time: *F*(2,2236) = 3.165, *p* = .042, $\:{\eta\:}_{p}^{2}$ = 2.82e-3 and confidence: *F*(2,173) = 18.151, *p* < .001, $\:{\eta\:}_{p}^{2}$ = 0.17). Post-hoc tests showed that inferences for events with overlapping ABC associations were more accurate and more confidently made for events encoded in the same compared with different contexts (accuracy: *t*(110) *=* 4.095, *p* < .001, *D* = 0.079; response times: *t*(2239) = 1.158, *p* = .479, *D* = 0.014; confidence: *t*(106) *=* 2.570, *p* = .031, *D* = 0.089). Also, inferences for events encoded in the same context were more accurate, faster, and more confidently made for events with overlapping ABC associations compared with events with non-overlapping XY associations (accuracy: *t*(110) *=* 7.395, *p* < .001, *D* = 0.118; response times: *t*(2240) *= -*2.513, *p* = .032, *D* = 0.032; and confidence: *t*(111) = 5.826, *p* < .001, *D* = 0.238). Finally, inference performance for events with overlapping ABC associations encoded in different contexts was more accurate and more confidently made compared with events with non-overlapping XY associations presented in the same context (accuracy: *t*(112) *=* 3.250, *p* = .004, *D* = 0.040; response times: *t*(2243) = 1.337, *p* = .375, *D* = 0.017 and confidence: *t*(111) = -3.148, *p* = .006, *D* = -0.149). A significant main effect of experiment was also observed in terms of accuracy and response times but not in terms of confidence (accuracy: *F*(1,112) = 17.224, *p* < .001, $\:{\eta\:}_{p}^{2}$ = 0.13; response time: *F*(1,110) = 6.816, *p* = .010, $\:{\eta\:}_{p}^{2}$ = 0.06; confidence: *F*(1,108) = 0.352, *p* = .554, $\:{\eta\:}_{p}^{2}$ = 2.34e-3), showing that inference performance was better in Experiment 2, that is when the encoding context was presented at retrieval.

**Fig. 4:**
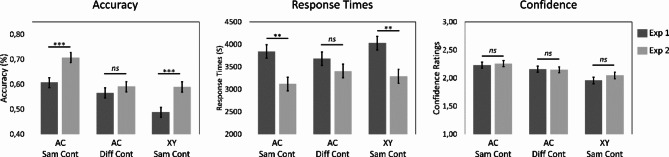
Mean accuracy, response times, and confidence ratings for the associative inference test in Experiment 1 (context absent at test) and Experiment 2 (context present at test). The error bars represent the standard errors of estimated marginal means. Statistical comparisons of interests are marked (*** for *p* ≤ .001; ** for *p* ≤ .01; * for *p* ≤ .05 and *ns* for *p* > .05).

Finally, a significant interaction between the two factors was observed for response times (accuracy: *F*(2,178) = 1.985, *p* = .140, $\:{\eta\:}_{p}^{2}$ = 0.02; response times: *F*(2,2236) = 7.628, *p* < .001, $\:{\eta\:}_{p}^{2}$ = 6.78e-3; confidence: *F*(2,173) = 0.546, *p* = .580, $\:{\eta\:}_{p}^{2}$ = 6.29e-3; see Fig. 4). This interaction shows that when context is presented at retrieval (i.e., Experiment 2), inferences for events encoded in the same context are faster compared with when context was absent (i.e., Experiment 1) (overlapping ABC events: *t*(141) = -3.260, *p* = .001, *D* = -0.133; and non-overlapping XY events: *t*(152) = -2.949, *p* = .004, *D* = -0.123). Interestingly, however, the context presentation at retrieval did not affect the response times for inferences across events with overlapping ABC associations but encoded in different contexts (*t*(147) = -1.058, *p* = .292, *D* = -0.044).

Even though no significant interaction was observed in terms of accuracy, we contrasted performance across the two experiments with t-tests, and Tukey corrected for multiple comparisons. This analysis revealed a similar pattern to the one found for response times. That is, inference performance across events encoded in the same context was higher when context was presented at the time of testing compared with when context was absent (overlapping ABC events: *t*(112) = 3.383, *p* = .001, *D* = 0.100; and non-overlapping XY events: *t*(108) = 3.810, *p* < .001, *D* = 0.101); however, inference accuracy across events with overlapping ABC associations but encoded in different context did not differ between the two experiments (*t*(112) = 0.799, *p* = .426, *D* = 0.026; see Fig. 4).

### Direct associations

In this final analysis, the performance for the direct associations was compared across the two experiments. The results revealed a significant main effect of association type, indicating lower memory performance for overlapping AB/BC pairs compared to non-overlapping XY pairs (accuracy: *F*(1,13090) = 32.358, *p* < .001, $\:{\eta\:}_{p}^{2}$ = 2.47e-3; response times: *F*(1,10868) = 31.937, *p* < .001, $\:{\eta\:}_{p}^{2}$ = 2.93e-3; and confidence: *F*(1, 11622) = 17.940, *p* < .001, $\:{\eta\:}_{p}^{2}$ = 1.54e-3). Additionally, an effect of context was found in the accuracy analysis, showing that participants were worse at retrieving pairs encoded in the same context (i.e., shared with another pair of associates) across the two learning phases (accuracy: *F*(1,13090) = 11.839, *p* < .001, $\:{\eta\:}_{p}^{2}$ = 9.04e-4; response times: *F*(1,10868) = 0.356, *p* = .551, $\:{\eta\:}_{p}^{2}$ = 3.28e-5; and confidence: *F*(1,11622) = 0.655, *p* = .418, $\:{\eta\:}_{p}^{2}$ = 5.64e-5).

The interaction between the two factors was significant for accuracy and confidence (accuracy: *F*(1,13090) = 6.008, *p* = .014, $\:{\eta\:}_{p}^{2}$ = 4.59e-4; response times: *F*(1,10868) = 1.905, *p* = .168, $\:{\eta\:}_{p}^{2}$ = 1.75e-4; and confidence: *F*(1, 11622) = 9.513, *p* = .002, $\:{\eta\:}_{p}^{2}$ = 8.18e-4). The post-hoc comparisons showed that performance for non-overlapping XY associations was better when the pairs were encoded in different contexts across the two learning sessions than when the pairs were encoded in the same context (accuracy: *t*(13096) = 4.499, *p* < .001, *D* = 0.038; confidence: *t*(11628) = 3.004, *p* = .003, *D* = 0.041). However, there were no differences of context for the overlapping AB/BC pairs (*p*s > 0.136).

No main effects of experiment nor second-order interactions involving the factor experiment were found (*p*s > 0.132). However, the third-order interaction was significant for the confidence analysis (*F*(1, 11622) = 4.653, *p* = .031, $\:{\eta\:}_{p}^{2}$ = 4.00e-4). The post-hoc analyses revealed that confidence for overlapping AB/BC pairs encoded in different contexts across the two learning sessions was higher in Experiment 1 compared with Experiment 2 (*t*(167) = 2.434, *p* =. 016, *D* = 0.116); however, for the other conditions, confidence was comparable across experiments (*p*s > 0.239).

### Summary

This analysis shows that presenting the context at the time of testing benefited associative inference performance across events encoded in the same context. This was the case for events with overlapping ABC associations as well as for events with non-overlapping XY associations. However, presenting the context at the time of testing did not benefit performance for inferences across events with different contexts, indicating that the context associated with only one of the relevant paired associates is not sufficient to improve inferences across events. Interestingly, however, no systematic differences were observed across the two experiments in memory performance for the direct associations. Altogether, these data suggest that context promotes the flexible retrieval processes involved in making inferential associations across events.

## General discussion

This study examined the role of episodic context in memory integration using an adapted version of the associative memory inference paradigm^6–8^. Our results showed that making inferences across events encoded in the same context is facilitated, but only when the context is present at the time of testing, suggesting that context promotes associative inference by providing privileged access to the events associated with that context.

Episodic memory is context dependent, and it is well documented that revisiting the encoding context at the time of test improves episodic remembering^15,18^. This effect is usually explained by the encoding-specificity principle, which suggests that the greater overlap between the encoding and retrieval, the better the episodic remembering^19^. Our study demonstrates that the privileged access provided by revisiting the encoding context can be utilized to recombine information from previous contextually associated events, thereby facilitating the associative inferential links between them.

Associative inferences across events can be accomplished by integrating, in a single memory representation, elements from past memories with elements from new events at the time of encoding^9^ and/or by retrieving and flexibly recombing past events during retrieval and by demands^10^. Both accounts are supported by neuroimaging data^7,8^, and the conditions promoting one mechanism over the other have been extensively researched^3^. For example, task demands^43^ and the temporal proximity of events^44^ influence the likelihood of past events becoming integrated with new events at the time of encoding. Here, we investigated the role of episodic context in the processes underlying making inferences across events. Our data showed that context could not enable the formation of an integrated memory representation during encoding. Instead, we observed that context promotes the flexible retrieval mechanisms at play during the inference testing. The context reinstatement at test promotes retrieval of the events associated with that context, enabling their flexible recombination and the creation of associative inferences across them. This idea also aligns well with the observation that context presentation at test improved associative inference for both events with and without overlapping elements, as long as they were encoded in the same context.

Interestingly, presenting context at test did not improve associative inference performance for events encoded in different contexts. This is somewhat surprising given that the context presented was associated with one of the relevant paired associates. One might expect that the presentation of the context during retrieval would activate the associated event, thereby facilitating associative inference. However, this was not the case. Instead, the context at retrieval did not enhance associative inference for events encoded in different contexts. This suggests that while context may help access events linked to the same context, it might simultaneously inhibit access to events tied to other contexts. As a result, when participants are required to make associative inferences across events encoded in different contexts, presenting a context at test provides no benefit. This idea aligns with recent theoretical frameworks regarding the role of context in episodic memory^26^.

Our results partially contradict the recent findings by Cox and colleagues^26^, who suggest that inference performance for events encoded in the same context improves when context is absent at test but not when it is present. However, this discrepancy is likely related to the different context manipulations employed in the studies. In Cox et al.^26^, each pair of overlapping associates was presented in a unique episodic context, whereas in our study, multiple paired associates were encoded within the same context. The unique association between a context and a given associate pair increases the diagnostic value of the context^45^, thereby enhancing the likelihood of retrieving associated past events when revisiting a context^22^. Consequently, in Cox et al.’s study^26^, revisiting a familiar context during encoding likely prompted the retrieval of the associated prior event, facilitating the integration of past and new elements into a compound memory representation. As a result, the retrieval of AC pairs may have relied on this integrated memory, even in the absence of the context. Furthermore, in Cox et al.’s study^26^ the direct associations were tested before the associative inference, which may influence the processes involved in making associative inferences and, consequently, the role of the context.

However, contemporary neurobiological models of memory conceive episodic memories arising from discrete events being bound to slowly drifting contexts^17,28–31^. Thus, in our study, we associated the episodic context with more than one paired associate, reducing its diagnostic value but allowing us to differentiate the role of the shared context from the role of the shared elements across events^16,33^. We found comparable inference performance for events encoded in the same and in different contexts, when context was absent at test (Experiment 1), while a benefit of encoding events in the same context was only observed when context was present at test (Experiment 2).

Additionally, we observed that memory performance for the direct associations was lower when associations were encoded in the same context (i.e., a context that was shared with another pair of associates) across the two learning sessions compared with different contexts. This corroborates previous findings^22,23^ and supports the idea that overlaps in context may induce memory interference due to competitive retrieval^21^. This finding is consistent with the fan effect^24,25^, which posits that as more associations are encoded within the same context, the number of competing memory traces increases. Consequently, the cognitive demand to resolve interference and retrieve a specific associate grows, leading to greater difficulty in accurately recalling individual memories.

The context manipulation used here, where multiple memory traces are associated with a specific episodic context, may lead to heightened levels of memory interference^21^. An effective strategy to counteract such interference involves encoding similar memory traces as distinct, non-integrated representations^46–48^. It is plausible that, in response to the interference posed by the same encoding context, participants encoded the overlapping associations as separate memory traces, thereby diminishing the reactivation of related events during subsequent learning instances. Notably, the formation of an integrated memory representation, incorporating elements from both past and present events, may necessitate an optimal level of reactivation of past memories^12,43^. Indeed, studies have shown that while robust memory reinstatement can facilitate memory integration, moderate levels of reactivation lead to the encoding of distinct, non-integrated memory representations^49^. This observation elucidates why encoding events within the same context did not enhance associative inferential memory performance in Experiment 1 when the encoding context was absent at test.

The memory impairment observed for the direct associations encoded in the same context could also be attributed to the inference test. Previous literature has reported memory detriments for direct events following engagement in an inference task^50,51^, particularly when the inference test is successful^52,53^. However, a complementary analysis found no support for this potential trade-off between memory for the direct associations and associative inference. Instead, we noted that successful associative inference memory was associated with better memory for the direct events (data reported in the supplementary note 2 and 3).

## Conclusions

Our study provides important insights into the role of context in memory integration. Memory integration across events can be supported by both integrative encoding and/or flexible retrieval processes. By employing a context manipulation aligned with contemporary models of episodic memory^16,33^, our findings indicate that context primarily influences the flexible retrieval processes supporting memory integration. Specifically, encoding events in the same context benefits inference performance if the context is revisited during the test. This finding aligns with the role of context in organizing personal past experiences and facilitating episodic remembering^17,28^. Context grants privileged access to associated events, facilitating the flexible recombination of these experiences and promoting memory integration across those events.

Future studies could explore whether making inferences based on events associated with a given episodic context promotes the creation of an integrated memory representation after the AC inference test. That is, after making an inference judgement for events associated with the same context, a single integrated memory representation combining memories from past events could be formed. This prediction would align well with previous findings, showing that making inferences on demand is associated with the creation of an integrated memory representation^52–54^.

## Electronic supplementary material

Below is the link to the electronic supplementary material.

Supplementary Material 1

## Data Availability

The data presented in this manuscript and the code used for the analyses are openly available on Open Science Framework: https://osf.io/qwrud/?view_only=d2886653bec94911bfc24f36c1085063.
