# Supplementary material for: The effects of episodic context on memory integration
Source: Sci Rep. 2024 Dec 4;14:30159. doi: 10.1038/s41598-024-82004-7 (PMC11615038; doi:10.1038/s41598-024-82004-7)
Supplement: Supplementary file 1 — Supplementary Material 1 [file 41598_2024_82004_MOESM1_ESM.docx]

# **Supplementary Material**

# **Supplementary Note 1: Final models used to test the effects of context on associative inference and direct associations performance.**

To analyze the effect of episodic context on associative inferences and direct associations we run linear mixed models on accuracy, and confidence and response times of correct responses. For all statistical models, and to prevent model overfitting^1^, each model started with the least complexity, i.e., with participants as the single random intercept . The inclusion of other effects as random slopes was evaluated by looking at the model fitting. If the model fitting improved with the inclusion of a given random slope, the random slope was kept. The final equations for each model are displayed in the Supplementary Table 1.1. We also contrasted memory performance for Experiment 1 and 2. The final equations of these models can be consulted in the Supplementary Table 1.2.

**Supplementary Table 1.1.** Final equations for each model in Experiment 1 and 2.

| Experiment 1 |
| --- |
| Associative Inference |
| $\boldsymbol{accuracy}\left( \boldsymbol{AC inference} \right)\boldsymbol{\sim Context Conditions+intercept+}\left( \boldsymbol{intercept} \right\vert\boldsymbol{participant)}$ |
| $\boldsymbol{response time}\left( \boldsymbol{AC inference} \right)\boldsymbol{\sim Context Conditions+intercept+}\left( \boldsymbol{intercept} \right\vert\boldsymbol{participant)}$ |
| $\boldsymbol{confidence}\left( \boldsymbol{AC inference} \right)\boldsymbol{\sim Context Conditions+intercept+}\left( \boldsymbol{intercept} \right\vert\boldsymbol{participant)}$ |
| Direct Associations |
| $\boldsymbol{accuracy}\left( \boldsymbol{direct association} \right)\boldsymbol{\sim Context+Association Type+Context*Association Type+intercept+}\left( \boldsymbol{Association Type+Context*Association Type+intercept} \right\vert\boldsymbol{participant)}$ |
| $\boldsymbol{response time}\left( \boldsymbol{direct association} \right)\boldsymbol{\sim Context+Association Type+Context*Association Type+intercept+}\left( \boldsymbol{intercept} \right\vert\boldsymbol{participant)}$ |
| $\boldsymbol{confidence}\left( \boldsymbol{direct association} \right)\boldsymbol{\sim Context+Association Type+Context*Association type+intercept+}\left( \boldsymbol{intercept} \right\vert\boldsymbol{participant)}$ |

| Experiment 2 |
| --- |
| Associative Inference |
| $\boldsymbol{accuracy}\left( \boldsymbol{AC inference} \right)\boldsymbol{\sim Context Conditions+intercept+}\left( \boldsymbol{Context Conditions+intercept} \right\vert\boldsymbol{participant)}$ |
| $\boldsymbol{response time}\left( \boldsymbol{AC inference} \right)\boldsymbol{\sim Context Conditions+intercept+}\left( \boldsymbol{intercept} \right\vert\boldsymbol{participant)}$ |
| $\boldsymbol{confidence}\left( \boldsymbol{AC inference} \right)\boldsymbol{\sim Context Conditions+intercept+}\left( \boldsymbol{Context Condition+intercept} \right\vert\boldsymbol{participant)}$ |
| Direct Associations |
| $\boldsymbol{accuracy}\left( \boldsymbol{direct association} \right)\boldsymbol{\sim Context+Association Type+Context*Association Type+intercept+}\left( \boldsymbol{intercept} \right\vert\boldsymbol{participant)}$ |
| $\boldsymbol{response time}\left( \boldsymbol{direct association} \right)\boldsymbol{\sim Context+Association Type+Context*Association Type+intercept+}\left( \boldsymbol{intercept} \right\vert\boldsymbol{participant)}$ |
| $\boldsymbol{confidence}\left( \boldsymbol{direct association} \right)\boldsymbol{\sim Context+Association Type+Context*Association type+intercept+}\left( \boldsymbol{intercept} \right\vert\boldsymbol{participant)}$ |

**Supplementary Table 1.2.** Final equations used to contrast performance across Experiment 1 and Experiment 2.

| Experiment 1 vs. Experiment 2 |
| --- |
| Associative Inference |
| $\boldsymbol{accuracy}\left( \boldsymbol{AC inference} \right)\boldsymbol{\sim Context Condition+Experiment+Context Condition*Experiment+intercept+}\left( \boldsymbol{Context Condition} \right\vert\boldsymbol{participant*experiment)+}\left( \boldsymbol{intercept} \right\vert\boldsymbol{participant*experiment)}$ |
| $\boldsymbol{response time}\left( \boldsymbol{AC inference} \right)\boldsymbol{\sim Context Condition+Experiment+Context Condition*Experiment+intercept+}\left( \boldsymbol{intercept} \right\vert\boldsymbol{participant*experiment)}$ |
| $\boldsymbol{confidence}\left( \boldsymbol{AC inference} \right)\boldsymbol{\sim Context Condition+Experiment+Context Condition*Experiment+intercept+}\left( \boldsymbol{Context Condition+intercept} \right\vert\boldsymbol{participant*experiment)}$ |
| Direct Associations |
| $\boldsymbol{accuracy}\left( \boldsymbol{direct association} \right)\boldsymbol{\sim Context+Association Type +Experiment+Context *Association Type+Association Type*Experiment+Context*Experiment+Context*Association Type*Experiment+ intercept+}\left( \boldsymbol{intercept} \right\vert\boldsymbol{participant*experiment)}$ |
| $\boldsymbol{response time}\left( \boldsymbol{direct association} \right)\boldsymbol{\sim Context+Association Type +Experiment+Context *Association Type+Association Type*Experiment+Context*Experiment+Context*Association Type*Experiment+ intercept+}\left( \boldsymbol{intercept} \right\vert\boldsymbol{participant*experiment)}$ |
| $\boldsymbol{confidence}\left( \boldsymbol{direct association} \right)\boldsymbol{\sim Context+Association Type +Experiment+Context *Association Type+Association Type*Experiment+Context*Experiment+Context*Association Type*Experiment+ intercept+}\left( \boldsymbol{intercept} \right\vert\boldsymbol{participant*experiment)}$ |

# **Supplementary Note 2: Correct associative inferences are associated with higher memory for the direct events in Experiment 1.**

Previous studies have reported a tradeoff between associative inference performance and memory for the direct associations^2,3^, suggesting that inference performance comes with the cost of losing memory for the direct associations. Moreover, Carpenter and Schacter’s^4,5^ showed that this tradeoff is only evident when inference is successful but not when inference is unsuccessful, which was however debated in other literatues^6,7^. Thus, we conducted an exploratory analysis to investigate if memory for the direct associations differed as a function of inference success and if the encoding context moderated this potential effect.

To test if the encoding context interacted with the potential effects of inference success on the retrieval of the direct association, we ran linear mixed models on the accuracies, confidence and response times of correct direct associations, considering the factors Inference Success (Successful *vs.* Non-successful) and Context Condition (ABC Overlapping associations encoded in same context *vs.* ABC Overlapping associations encoded in different context *vs.* XY Non-overlapping associations encoded in same context). The models included participants as random intercepted. The inclusion of other effects as random slopes was evaluated by looking at the model fitting. If the model fitting increased with the inclusion of a given random slope, the random slope was kept (see supplementary table 2.1. for the final equation of each model).

We found significant effects of Inference Success (accuracy: *F*(1,4018) = 11.421, *p* < .001, $\eta_{p}^{2}$ = 2.83e-3; response times: *F*(1,3224) = 37.674, *p* < .001, $\eta_{p}^{2}$ = 0.01; and confidence: *F*(1,3232) = 26.577, *p* < .001, $\eta_{p}^{2}$ = 8.16e-3) indicating that retrieval of the direct associations was more accurate, faster and more confident when inference was successful compared with when inference was unsuccessful.

Additionally, the effect of context was also significant in terms of accuracy and response times (accuracy: *F*(2,3977) = 3.009, *p* = .046, $\eta_{p}^{2}$ = 1.55e-3; response times: *F*(2,3212) = 6.233, *p* = .002, $\eta_{p}^{2}$ = 3.87e-3; confidence: *F*(2, 3212) = 1.0737, *p* = .342, $\eta_{p}^{2}$ = 6.68e-4). The post-hoc tests indicated that participants were more accurate retrieving the XY non-overlapping associations compared with the ABC overlapping associations encoded in the same context (*t*(3982) = 2.44, *p* = .040, *D* = 0.036). However, accuracy performance was statistically comparable in all the other comparisons (*p*s > .238). Additionally, participants were faster at retrieving XY non-overlapping associations compared with ABC overlapping associations encoded in the same context (*t*(3218) = -3.12, *p* = .005, *D* = -0.066) and in different contexts (*t*(3218) = -2.90, *p* = .011, *D* = -0.060). However, the response time of overlapping ABC pairs encoded in same and different contexts was not statistically different (*t*(3218) = -0.30, *p* = .953, *D* = -0.006).

The interaction term was significant in the confidence analysis (accuracy: *F*(2,4009) = 2.591, *p* = .075, $\eta_{p}^{2}$ = 1.29e-3; response times: *F*(2,3220) = 0.633, *p* = .531, $\eta_{p}^{2}$ = 3.93e-4; and confidence: *F*(2,3225) = 4.849, *p* = .008, $\eta_{p}^{2}$ = 3.00e-3; see Supplementary Table 2.2.). Participants were more confident retrieving overlapping associations encoded in the same context and in different contexts when inference was successful compared with when inference was unsuccessful (same context: *t*(3236) = 4.219, *p* < .001, *D* = 0.142; different context: *t*(3232) = 4.132, *p* < .001, *D* = 0.134). However the retrieval confidence of XY non-overlapping associations was not affected by inference success (*t*(3229) = 0.503, *p* = .615, *D* = 0.016).

In summary, we observed that successful inferences were associated with better memory for the original direct associations^6,7^. Importantly, the encoding context did not seem to mediate this effect. The interaction between successful inference and context was significant in the confidence model, but it was driven by the fact that confidence was higher for successful compared with unsuccessful inferences for events with overlapping ABC associations compared with events with non-overlapping XY associations, independently of the encoding context condition. See Supplementary Table 2.2.

**Supplementary Table 2.1.** Final equations used for each model.

| Context as a function of Inference Success |
| --- |
| $\boldsymbol{accuracy}\left( \boldsymbol{direct association} \right)\boldsymbol{\sim Context Condition+ Inference Success+Context Condition*Inference Success+intercept+}\left( \boldsymbol{intercept} \right\vert\boldsymbol{participant)}$ |
| $\boldsymbol{response time}\left( \boldsymbol{direct association} \right)\boldsymbol{\sim Context Condition+ Inference Success+Context Condition*Inference Success+intercept+}\left( \boldsymbol{intercept} \right\vert\boldsymbol{participant)}$ |
| $\boldsymbol{confidence}\left( \boldsymbol{direct association} \right)\boldsymbol{\sim Context Condition+ Inference Success+Context Condition*Inference Success+intercept+}\left( \boldsymbol{intercept} \right\vert\boldsymbol{participant)}$ |

**Supplementary Table 2.2.** Mean accuracy, Response Times (RTs) and Confidence for the direct associations across context conditions and inference success in Experiment 1.

|  | | ACC | RT (ms) | Confidence |
| --- | --- | --- | --- | --- |
| Successful inference | |  |  |  |
|  | ABC overlapping associations encoded in the same context | 0.830 | 2475 | 2.72 |
|  | ABC overlapping associations encoded in different contexts | 0.819 | 2437 | 2.74 |
|  | XY non-overlapping associations encoded in the same context | 0.833 | 2261 | 2.69 |
| Non-successful inference | |  |  |  |
|  | ABC overlapping associations encoded in the same context | 0.751 | 2653 | 2.58 |
|  | ABC overlapping associations encoded in different contexts | 0.787 | 2695 | 2.60 |
|  | XY non-overlapping associations encoded in the same context | 0.820 | 2566 | 2.68 |

# **Supplementary Note 3: Correct associative inferences are associated with higher memory for the direct events in Experiment 2.**

In Experiment 2, we also tested if the encoding context interacted with the potential effects of inference success on the retrieval of the direct association. This was tested with linear mixed models run on the accuracies, response times and confidence of the direct associations. The model considered the factors Inference Success (Successful *vs.* Non-successful) and Context Condition (ABC Overlapping associations presented in the same context *vs.* ABC Overlapping associations presented in different context *vs.* XY Non-overlapping associations presented in the same context). See supplementary table 3.1. for the final equations of each model.

Significant effects of Inference Success were observed for response times and confidence (accuracy: *F*(1,3862) = 0.001, *p* = .976, $\eta_{p}^{2}$ = 2.30e-7; response times: *F*(1,3117) = 13.961, *p* < .001, $\eta_{p}^{2}$ = 4.46e-3; and confidence: *F*(1,51) = 9.840, *p* = .002, $\eta_{p}^{2}$ = 0.16), indicating that participants were faster and more confident at retrieving direct associations if the inference test was successful. The effect of context was significant for the response times and confidence measures (accuracy: *F*(2,3836) = 0.884, *p* = .413, $\eta_{p}^{2}$ = 4.61e-4; response times: *F*(2,3107) = 6.762, *p* = .001, $\eta_{p}^{2}$ = 4.33e-3; and confidence: *F*(2,2655) = 3.921, *p* = .020, $\eta_{p}^{2}$ = 2.74e-3). The XY non-overlapping associations were retrieved faster and more confidently compared with the ABC overlapping associations encoded in different contexts (response times: *t*(3113) = -3.647, *p* < .001, *D* = -0.052; confidence: *t*(2644) = 2.727, *p* = .018, *D* = 0.069). The other differences were not observed (*p*s > .088). The interaction term between the two factors was non-significant for all models (accuracy: *F*(2,3862) = 0.939, *p* = .391, $\eta_{p}^{2}$ = 4.86e-4; response times: *F*(2,3115) = 1.115, *p* = .328, $\eta_{p}^{2}$ = 7.16e-4; confidence: *F*(2, 3111) = 1.1439, *p* = .319, $\eta_{p}^{2}$ = 7.35e-4; see Supplementary Table 3.2.).

In summary, inference success was associated with better retrieval performance for the direct events, at least in terms of response times and confidence, corroborating what was previously found in Experiment 1.

**Supplementary Table 3.1.** Final equations used for each model.

| Context Condition as a function of Inference Success |
| --- |
| $\boldsymbol{accuracy}\left( \boldsymbol{direct association} \right)\boldsymbol{\sim Context Condition+ Inference Success+Context Condition*Inference Success+intercept+}\left( \boldsymbol{intercept} \right\vert\boldsymbol{participant)}$ |
| $\boldsymbol{response time}\left( \boldsymbol{direct association} \right)\boldsymbol{\sim Context Condition+ Inference Success+Context Condition*Inference Success+intercept+}\left( \boldsymbol{intercept} \right\vert\boldsymbol{participant)}$ |
| $\boldsymbol{confidence}\left( \boldsymbol{direct association} \right)\boldsymbol{\sim Context Condition+Inference Success+Context Condition*Inference Success+intercept+}\left( \boldsymbol{Inference Success+intercept} \right\vert\boldsymbol{participant)}$ |

**Supplementary Table 3.2.** Mean accuracy, response times (RTs) and Confidence for the direct associations across context conditions and inference success in Experiment 2.

|  | | ACC | RT (ms) | Confidence |
| --- | --- | --- | --- | --- |
| Successful inference | |  |  |  |
|  | ABC overlapping associations encoded in the same context | 0.812 | 2239 | 2.65 |
|  | ABC overlapping associations encoded in different contexts | 0.805 | 2309 | 2.62 |
|  | XY non-overlapping associations encoded in the same context | 0.818 | 2208 | 2.65 |
| Non-successful inference | |  |  |  |
|  | ABC overlapping associations encoded in the same context | 0.789 | 2387 | 2.57 |
|  | ABC overlapping associations encoded in different contexts | 0.823 | 2450 | 2.50 |
|  | XY non-overlapping associations encoded in the same context | 0.824 | 2244 | 2.60 |

# **References**

1. Matuschek, H., Kliegl, R., Vasishth, S., Baayen, H. & Bates, D. Balancing Type I error and power in linear mixed models. *J. Mem. Lang.* **94**, 305–315 (2017).

2. Banino, A., Koster, R., Hassabis, D. & Kumaran, D. Retrieval-based model accounts for striking profile of episodic memory and generalization. *Sci. Rep.* **6**, 31330 (2016).

3. L Varga, N., Gaugler, T. & Talarico, J. Are mnemonic failures and benefits two sides of the same coin?: Investigating the real-world consequences of individual differences in memory integration. *Mem. Cognit.* **47**, 496–510 (2019).

4. Carpenter, A. C. & Schacter, D. L. Flexible retrieval: When true inferences produce false memories. *J. Exp. Psychol. Learn. Mem. Cogn.* **43**, 335–349 (2017).

5. Carpenter, A. C. & Schacter, D. L. False memories, false preferences: Flexible retrieval mechanisms supporting successful inference bias novel decisions. *J. Exp. Psychol. Gen.* **147**, 988–1004 (2018).

6. Boeltzig, M., Johansson, M. & Bramão, I. Ingroup sources enhance associative inference. *Commun. Psychol.* **1**, 40 (2023).

7. de Araujo Sanchez, M. A. & Zeithamova, D. *Cognition* **234**, 105385 (2023).
